# Mechano-Immunomodulation in Space: Mechanisms Involving Microgravity-Induced Changes in T Cells

**DOI:** 10.3390/life11101043

**Published:** 2021-10-03

**Authors:** Sarit Dhar, Dilpreet Kaur Kaeley, Mohamad Jalal Kanan, Eda Yildirim-Ayan

**Affiliations:** 1 Department of Bioengineering, College of Engineering, University of Toledo, Toledo, OH 43606, USA; sarit.dhar@rockets.utoledo.edu (S.D.); dilpreet.kaeley@rockets.utoledo.edu (D.K.K.); mohamad.kanan@rockets.utoledo.edu (M.J.K.); 2 Department of Orthopaedic Surgery, University of Toledo Medical Center, Toledo, OH 43614, USA

**Keywords:** microgravity, T cells, altered gravity, cell signaling, immunity, space, immune response, weightlessness, apoptosis, activation

## Abstract

Of the most prevalent issues surrounding long-term spaceflight, the sustainability of human life and the maintenance of homeostasis in an extreme environment are of utmost concern. It has been observed that the human immune system is dysregulated in space as a result of gravitational unloading at the cellular level, leading to potential complications in astronaut health. A plethora of studies demonstrate intracellular changes that occur due to microgravity; however, these ultimately fall short of identifying the underlying mechanisms and dysfunctions that cause such changes. This comprehensive review covers the changes in human adaptive immunity due to microgravity. Specifically, there is a focus on uncovering the gravisensitive steps in T cell signaling pathways. Changes in gravitational force may lead to interrupted immune signaling cascades at specific junctions, particularly membrane and surface receptor-proximal molecules. Holistically studying the interplay of signaling with morphological changes in cytoskeleton and other cell components may yield answers to what in the T cell specifically experiences the consequences of microgravity. Fully understanding the nature of this problem is essential in order to develop proper countermeasures before long-term space flight is conducted.

## 1. The Importance of Human Health and Immunity for Space Exploration

As we enter the new decade, humans are as focused as ever on the exploration of space. The rationale of such a profound goal lies in the potential for long-term colonization on planets besides Earth. The National Aeronautics and Space Administration’s (NASA) “Artemis” program aims to reach the Moon again by 2024, which is the first step to landing a human on Mars [1]. This would require human astronauts to potentially live in space for years, something that has not been attempted to this day. Such an endeavor comes with perplexing problems including a lack of sufficient knowledge on how the human body adapts and reacts to the microgravity environment. Thus, preparing for possible biological and psychological complications caused by deep-space exploration is a priority for space research programs.

From the days of the first Apollo missions to the moon, it has been observed that astronauts suffer from physiological changes due to the challenging environment in space, with which they are unacquainted [2,3,4]. Such changes include, but are not limited to, bone density loss [5,6,7], muscle atrophy [8,9,10], impaired eyesight [11], cardiac dysfunction [12], loss of proprioception [13], immune dysregulation [14,15], and changes in the expression of many genes [16,17]. These changes can be attributed to numerous factors in space such as radiation, disrupted circadian rhythm, and most of all the lack of Earth’s gravity, which has played a pivotal role in determining baseline development and homeostasis since the beginning of human evolution.

The gravity environment in space is termed “microgravity” (μG) and is defined as approximately 10^−6^ of Earth’s surface gravity (G), as there is never truly a complete absence of gravity [18]. The effects of μG on various cell types have been documented in bone, cartilage, and endothelial cells, to name a few, and immune cells are no exception [5,6,19,20]. Among the various microgravity-induced side effects, a compromised or altered immune response can have serious consequences and jeopardize the survival of humans in space. Therefore, understanding how μG affects the functions and components of the immune system over short or long periods is very crucial.

The human immune system is a complex interdisciplinary network that incorporates a wide host of cells and molecules which maintain the organism’s health. It serves to protect the human body against malignant tissues and exogenous factors such as pathogens. It is broken into two branches: innate and adaptive immunity. Innate immunity does not require prior exposure to a pathogen to activate a swift and nonspecific response, whereas adaptive immunity requires previous exposure to a pathogen in order to initiate a specific response [21]. Innate immunity includes the physical barriers of the skin and mucous membranes that prevent the entry of pathogens into the body. The innate immune system also includes defensive cells that protect the body against pathogens that pass the barriers. These cells include natural killer cells, dendritic cells, neutrophils, eosinophils, basophils, mast cells, and the monocyte/macrophage system. Adaptive immune response occurs in conjunction with the innate immune system in order to eliminate pathogens. It is made up of T lymphocytes, B lymphocytes, antibodies in the blood, and messenger cytokines in the blood and tissue [22]. In this comprehensive review paper, we will focus on T cells.

Although it is known that the immune response is affected by μG [23,24,25], the cellular mechanisms of such dysregulation have not been fully elucidated. The causes and mechanisms by which cells behave as they do in a μG environment are still largely unknown. We will investigate the phenomenon from a mechanobiological perspective, as the cells of the immune system are subjected to mechanical unloading in the absence of gravity. This mechanical sensitivity is best seen in how it affects signal cascades and pathways. Signals are important for cell communication and utilized by countless cell types [26], and the subtraction of gravitational forces may be perceived as an extracellular stressor [27]. In turn, the signals produced affect gene expression, protein composition, cytoskeletal arrangement and cell activity. Alterations in mechanosensitive signaling can severely affect the function of the human immune response at the cellular level and are indicated to be sensitive to gravitational unloading [28,29]. Thus, in this comprehensive review, we will detail the conclusions of studies regarding gravisensitive T cell signaling in a μG environment. The mechanobiological study of this important immune cell may shed some light on its observed dysregulation in space.

## 2. Studying Cellular Responses to Altered Gravity

While prolonged space missions with astronauts are the most accurate method of understanding the effects of μG on immune cells, the cost of missions is extremely high and this poses limitations on the choice of experimental parameters, including the experiment duration and number of test subjects. Another accurate means of demonstrating the effects of μG on immune cells is conducting in vitro experiments [30,31]. However, this method is also very costly and not accessible to most researchers [32]. Furthermore, space flight studies demonstrate that cells adapt to changes in μG over time. This means that the cellular response is transient. Thus, it is important to run longitudinal studies in a μG environment in order to identify early adaptive changes and understand whether these changes persist during long-term μG exposure. Aside from experiencing μG astronauts are exposed to other stressors which may affect their immune system, including radiation, constant fluid shifts, altered circadian rhythms, prolonged isolation and confinement, and stressful task schedules [14]. Thus, understanding the sole effect of μG on cellular responses is not possible in space flight studies.

### 2.1. Microgravity Analogs

To address challenges associated with space flight studies (such as reducing cost, increasing accessibility, and improving repeatability), simulated μG platforms have been utilized to mimic the effects of μG or weightlessness on Earth [33,34]. It should be noted that several review articles already have explained simulated μG platforms [35,36,37,38]. Thus, in this review paper, we briefly mention these platforms to lay a foundation of knowledge and introduce the terminology for the rest of the review paper.

*Hindlimb suspension (HLS)*, or *hindlimb unloading (HU)*, can be used on rodents to mimic the effects of μG by elevating the hind limbs and maintaining a head-down tilt position at varying angles, causing a fluid shift towards the head of the animal [39,40]. This model skeletally unloads the hind limbs of rodents, mimicking immunological changes associated with spaceflight [41,42]. HLS serves as a useful model for in vivo experiments on rodents but displays clear drawbacks in that these experiments cannot be conducted on humans.

*Head down bed rest (HDBR)* is used on humans to mimic μG and allows in vivo experiments on Earth in an attempt to produce countermeasures for immunological changes in long-term spaceflight. Subjects in these experiments lay flat in a head-down position at −6° for up to 120 days [43,44]. Although varying data have been collected from HDBR studies, it is believed to be the most practical model for examining multi-system responses of simulated μG in humans, as the subjects can be monitored and examined easily [45,46,47,48,49]. Variations in results could be due to the varying lengths of the studies and the environment in which the studies were conducted.

*Two-dimensional (2D) clinorotation* is a popular μG simulation method as it is simple to set up and cost effective. It is achieved by fixing in vitro experiments to a clinostat that rotates slowly, usually under 60 rpm, along a longitudinal axis parallel to the ground [50,51,52,53,54,55]. This does not achieve true μG but counteracts the sedimentation effect of molecules, mimicking the effect of true μG.

*Rotary wall vessels (RWV)* utilize a similar mechanism to the 2D clinostat to imitate the effects of microgravity [56]. The RWV includes a closed bioreactor in which cells are suspended and exposed to simulated μG [25,57,58,59,60,61,62,63]. A complication of this technique is that suspended cells may experience additional shear forces if air bubbles are present within the bioreactor, or if the bioreactor apparatus is rotated at a faster speed [64,65]. Alternatively, experiments utilizing adherent cells may compromise the samples if care is not taken when detaching them from the vessel walls. A benefit of the RWV is that the cell culture is in an enclosed capsule and media can be introduced or removed through built-in ports without disturbing the cells or jeopardizing the experiment [66].

*Three-dimensional (3D) clinostat*, also known as a *random positioning machine (RPM)*, rotates in two planes simultaneously, increasing the random positioning effect [36]. Three-dimensional clinostats consist of two planes aligned perpendicularly, one inside of the other, with two separate motors rotating at random speeds in each plane to randomize the gravitational vector [63,67,68,69,70,71]. The second plane of rotation further decreases the sedimentation effect, improving upon the 2D clinostat [72]. One major pitfall of 3D clinostats is the absence of a gas-exchange tube due to the two-frame design, which may be important to some experiments [38].

### 2.2. Microgravity Platforms

*Parabolic flight (PF)* is a method of exposing experimental setups to real μG on Earth. This includes loading experimental samples or subjects on an aircraft and flying a parabolic path which exposes those aboard the aircraft to 20 s intervals of weightlessness [55,73,74,75,76,77]. Between the intervals of weightlessness, hyper-gravity is experienced as the aircraft accelerates upward to create the next parabolic flight path. Careful protocols must be drawn out in order to control for the hyper-gravity intervals, which may adversely affect the experiment [36]. Parabolic flights are best suited for experiments analyzing the short-term effects of μG [78]. Although it is a viable method of μG exposure, the short weightlessness intervals along with the inevitable hyper-gravity exposure and cost of flying cause PF to be less than ideal for some experiments.

*Suborbital ballistic rockets*, also known as *sounding rockets (SR)*, are able to achieve true μG for a relatively short period, though longer than PF. Sounding rockets consist of an engine and an experimental payload that is propelled into the sky along a predetermined flight path, then falls towards the Earth once the engine(s) have separated [53,77,79,80,81]. The TEXUS-49 sounding rocket experiment was able to achieve suborbital μG with a quality of 10^−5^ G for 378 s [82]. Similar to PF, the payload of an SR experiences an interval of hyper-gravity during its ascent which must be controlled for.

*Spaceflight* experiments on the ISS or unmanned orbital spacecrafts appear to be the most accurate in studying the effects of μG [83]. A great benefit of spaceflight experiments is the capability of running in vivo experiments on living organisms while exposing them to all of the effects experienced during spaceflight [16,84,85,86]. In vitro spaceflight experiments may include hardware that is flown to space and run aboard the ISS, which has in-space controls such as a clinostat aboard the spacecraft in addition to the ground controls [31,87]. The greatest downside to these experiments is the cost associated with sending astronauts and experimental setups to space.

Important scientific discoveries were made on how T cells react to the altered gravity environment using aforementioned microgravity analogs or true microgravity platforms. Table 1 tabulates some of these prominent studies that investigated how the expression of important pro-and anti-inflammatory markers changes when T cells from various sources are exposed to simulated or real microgravity.

The detailed information in Table 1 demonstrates that T cell response depends not only on the source of the T cells but also on how long they are exposed to microgravity and how microgravity is created. Now that we understand the experimental background of μG studies, and that T cells respond to altered gravity, the next important research question is: what are these specific changes, and the mechanisms behind them? We will investigate the inter- and intra-cellular processes on a molecular level to determine any gravisensitive steps within T cell immunity.

## 3. Mechanisms Involving Microgravity-Induced Changes in T Cell Signaling Pathways

Cell signaling is perhaps the most important method of communication in the body [106,107]. Signal transduction is a part of nearly every cell and mediates everything from cellular production to life cycle [26]. Through a series of molecules and receptors, signals can directly alter genetic expression and therefore cell function. Cells take cues from their neighboring cells and the environment to determine what they do and how they do it. A system so perceptive is also just as sensitive; thus, changes of the upstream signal may lead to large chain reaction responses [108]. For this reason, signal transduction is an intriguing subject of study with regard to μG. An extreme and unpredictable change such as the removal of gravity-induced loading may exert its overall effects through cell signaling pathways. T cells were chosen as the topic of investigation because of their large role in adaptive immunity. These lymphocytes are directly responsible for the adaptive immune response, an important process in keeping humans protected from repeat exposure. A review of current literature revealed that innate immune signaling, modeled by macrophages, has recently been investigated in microgravity [109]. Additionally, there is little experimental evidence about the effect of μG on other immune cell types, such as Natural Killer Cells. Currently, there has not been an extensive review of specific mechanistic changes in adaptive T cell signaling in μG such as these.

T cells recognize and destroy infected cells by using special surface structures that can bind specific pathogens. T cells also have the ability to remember previous antigens; this allows for an efficient response to known antigens. In the body, T cells have a number of functions, from killing infected cells to acting as messengers when recruiting more immune cells to respond to a stimulus. During a response, T cells can develop into specialized cells such as helper T cells, cytotoxic T cells, memory T cells, and regulatory T cells [22]. It is important to note that some studies show variable results in experiments performed under μG conditions on T cell precursors [110,111], on populations of these specialized T cells [44,49,105,112,113], and on the distribution of T cells in the body [103,114]. However, most of the studies that we investigated have broadly used T lymphocytes as a model for adaptive immunity, obtained from a variety of sources. To understand adaptive immunity in μG, the signaling pathways regarding T cells should be considered. Specifically, activation and apoptotic pathways of T cells are especially important in understanding general adaptive immune functions.

**Figure 1:**
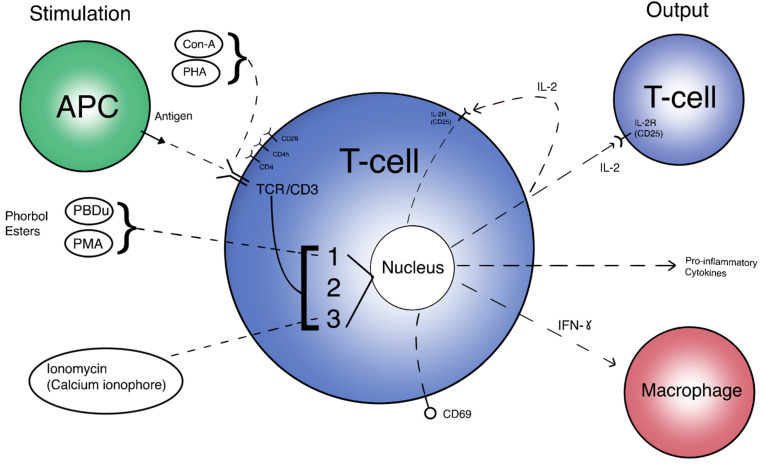
A simplified view of T cell activation. Inputs are different methods of stimulation and their respective targets. Outputs are expressed proteins and molecules targeting other immune cells. 1 = PKC/NF-kB pathway. 2 = Ras/AP-1 pathway. 3 = Calcineurin/NFAT pathway.

The T cell activation pathway can begin by two general means in vivo: direct interaction with an Antigen-Presenting Cell (APC), and activation via Interleukin-2 (IL-2). Although most μG studies use antigens such as phorbol myristate acetate (PMA), phytohaemagglutinin (PHA), or Concanavalin A (ConA) as stimulation methods, there are a few that review APC interactions via dendritic cells or via the effects of the monocyte/macrophage system on T cells by looking at levels of tumor necrosis factor alpha (TNF-a) [62,71,76,86]. Direct activation requires the presented antigen to form a complex with the naive T cell Receptor (TCR), along with CD28 co-stimulation and cytokine signaling. Alternatively, IL-2 secretion from the initial cell may begin the cascade of auto-proliferation and proliferative signaling of other T cells [115]. This delicate activation pathway may very well be changed by μG and significantly affect immune regulation. Figure 1 displays the T cell “flowchart”, an overall scheme to compare inputs and outputs of T cell activation. Different stimulation methods (inputs) and their targets can be seen on the left side of the image, while activation responses (outputs) are depicted on the right side. The initial T cell is directly activated and begins an amplificatory, proliferative cascade to other T cells through IL-2, among other pro-inflammatory cytokines. This amplification reaction also includes macrophages and other immune cells to complete the immune response.

Normal activation is needed for proper adaptive immune defense; lack of T cell activation leads to impaired immune response. The consequences of impaired immunity include infection by pathogens and possibly unchecked neoplastic growth, which may lead to illness and potentially death. Apoptosis is also an important factor in T cell immunity, as pre-planned cell death lends itself to immune system efficiency. Specific cell death pathway alterations due to μG may affect the overall normal function of T cells.

### 3.1. Signaling Pathways Responsible for T Cell Activation under Microgravity

It has been well documented that T cell activation and its proliferation are downregulated in μG [52,64,66,88,90,100]. As such, investigations primarily aim to uncover the interruption in cell signaling that leads to the limited activation levels in T cells. One of the most identifiable intercellular signaling molecules is IL-2, a cytokine essential for early T cell activation. IL-2 is the most widely accepted and commonly used method to test T cells for activation response, dating back to early studies. It acts as a direct measure of T cell activation, production, and signal amplification. While other pro-inflammatory cytokines may also be tested, none are as directly tied to the T cell activation cascade as IL-2, and therefore not as used in the literature of interest. Many studies have demonstrated that IL-2 production and the IL-2 receptor (IL-2R/CD25) are decreased in real and simulated μG conditions [46,51,52,64,67,68,69,88,90,97,101,116]. For instance, Chang et al. conducted experiments on the space shuttle Discovery and showed that IL-2 secretion in mice was decreased after a 15-day long μG exposure as compared to counterparts on Earth [101]. The decrease in IL-2 suggests that the in-flight mice have depressed T cell activation response in the absence of gravity. Besides in-flight studies, the studies conducted under a simulated gravity environment demonstrated decreased IL-2 expression. Boonyaratanakornkit et al. studied T cells isolated from peripheral blood leukocytes using 3D clinostat. They demonstrated that 4 h of μG exposure decreased IL-2 expression [67]. Another study conducted with RWV for 24 h proved that μG decreased expression of IL-2 in both CD4+ and CD8+ T cells [64]. Interestingly, CD4+ T cells were more sensitive to μG changes than CD8+ T cells, as evidenced by IL-2 levels. Taken together, it is reasonable to assume μG has a suppressive effect on IL-2 in the T cell activation pathway.

IL-2R/CD25 is just as important as IL-2 in determining the efficacy of T cell activation. The expression of cell surface receptors directly influences downstream pathways. IL-2R is shown to be downregulated in both real and simulated μG via parabolic flight and 2D clinostat, respectively [51]. In both cases, T cells activated with Con-A and anti-CD28 showed rapid suppression of IL-2 in μG. In addition, testing of CD3 (a very early-pathway cell surface receptor) expression also showed evidence of downregulation in μG. Decreased expression of TCR/CD3, or any of its subunits, on the cell surface would lead to significant impairment of the activation signaling cascade in T cells, as it is one of the first steps that initiates activation. This suggests that T cell activation receptors are inhibited throughout the pathway. Similarly, in an aforementioned study, IL-2R expression was confirmed to be downregulated in μG after Con-A anti-CD28 activation [67]. IL-2R was again shown to be downregulated in μG via 2D clinostat and shuttle spaceflight after activation by combinations of PMA, Leu4, PHA, and ionomycin [52]. Both modeled μG and real μG under the several listed stimulation methods proved the wide range and effect of μG on T cell activation, as shown by depressed IL-2R and CD69 levels. In a study by Martinez et al., both lL-2 and IL-2R were confirmed to be decreased in activated T cells in μG as well [69]. The downregulation of IL-2 paired with decreased IL-2R/CD25 receptor expression contributes to the depressed activation and proliferation of T- lymphocytes.

**Figure 2:**
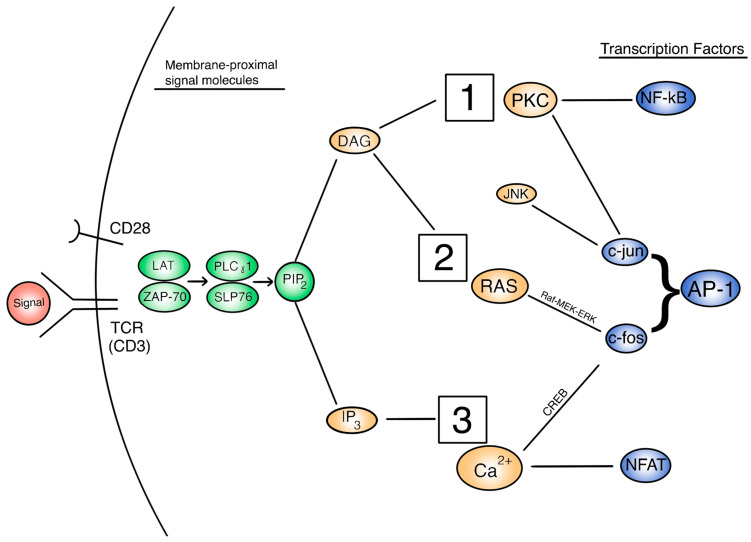
A view of the intracellular T cell activation signal cascade, with signal molecules of interest. 1 = PKC/NF-kB pathway. 2 = Ras/AP-1 pathway. 3 = Calcineurin/NFAT pathway.

The expression of IL-2 and its receptor are indicative of activated T cells later in the activation pathway. However, the regulation of IL-2 and other cytokine signals are controlled at some upstream signaling pathways [26]. Thus, in order to understand how IL-2 secretion is affected and this subsequently affects T cell activation, we need to focus on the three important signaling pathways: PKC/NF-kB, Ras/AP-1, and Calcineurin/NFAT. Figure 1 depicts a broad, extracellular look at these pathways and how they are activated in the overall function of the activated T cell. Figure 2 provides a more detailed look at the relationship between these pathways intracellularly. Each activation pathway works in conjunction with the others and ends in a transcription factor that helps to express relevant proinflammatory genes (such as IL-2 and other cytokines, seen in Figure 1 outputs). Inhibition or dysregulation at any point in these pathways, or upstream from them will be responsible for IL-2 reduction and the subsequent suppression of T cell activation in μG. We will investigate which steps in these pathways may be impacted by microgravity.

#### 3.1.1. PKC/NF-kB Pathways

First, the family of NF-kB pathways, specifically p65 and p50, are known players in T cell activation pathways and can determine the production of IL-2 [26]. In both simulated and real μG, NF-kB levels and activity were shown to decrease under the conditions compared to control [85,90]. Transcription factors p65 and p50 were both shown to be affected by μG in a T cell genomic study [80]. Under real μG provided on the ISS, T cells activated by Con-A and anti-CD28 had significant inhibition of p65 and p50 expression. In a comprehensive study, Paulsen et al. showed the overall effect of μG on key signal transduction molecules [50]. In μG provided by a 2D clinostat for 5 min, it was found that nuclear translocation of p65 (an NF-kB transcription factor) was significantly reduced in both stimulated and unstimulated T cells. The reduction of p65 function may directly correlate to a reduction in IL-2 and activation cascade signaling. Looking further upstream of this NF-kB pathway, PKC should be examined as a possible culprit for gravisensitive dysfunction.

In an earlier discussed study, PMA was shown to restore IL-2R expression in μG-inhibited T cells in 2D clinorotation; this suggests that a signaling step at PKC, or upstream from it, is directly affected by μG associated inhibition of T cell activation pathways, since PMA directly activates PKC [52]. In RWV μG treatment for 72 h, T- lymphocytes showed a decrease in PKC activation and cellular locomotion [65,90]. The activity of PKC was restored upon stimulation via PMA, corroborated by the previous study as well. The initial decrease of PKC activity in μG may affect the downstream NF-kB (p65) pathway and IL-2 expression. Although the time of these experimental treatments should be kept in mind, it seems that the PKC-mediated NF-kB T cell activation pathway is significantly affected by μG. In an RWV study by Cooper and Pellis, it was observed that T cell activation was initially suppressed in μG after full activation at the TCR by PHA [66]. In turn, it was observed that upon direct PKC activation by PMA, activation marker levels were restored even under simulated μG. When Ca^2+^ was also directly stimulated by ionomycin with PMA, activation was similarly restored. PKC and Ca^2+^ pathways are activated by diacylglycerol (DAG) and inositol trisphosphate (IP3), respectively, which come from the cleavage of the precursor molecule phosphatidylinositol biphosphate (PIP2). Knowing that the Ca^2+^ pathway may be intact during the initial PHA stimulation, IP3 must be present, and it can be assumed that DAG is also present as they both come from PIP2. This indicates that there is dysfunction in the interaction between DAG and PKC, as PKC downstream molecules are restored by direct PMA activation. Results from other studies concur that the inhibition of the PKC signaling pathway occurs in a signaling step downstream from the surface receptors and upstream of PKC itself [63,68,117].

Looking between TCR membrane activation and PKC activation, Tauber et al. found that ZAP-70, an important signaling molecule immediately subsequent to TCR and upstream of PKC, is decreased after 5 min of 2D clinostat-provided μG [51]. Simons et al. investigated the involvement of signaling molecules upstream of DAG in antibody-activated CD4+ T cells using an RWV for simulated μG [118]. They tested key TCR-proximal molecules within the activation pathway such as ZAP-70, PLCy1, and SLP-76, among others. The data showed no significant difference between the levels of the investigated molecules upstream of PKC in μG vs. 1G. In contrast to the beliefs of previous studies mentioned, they concluded that the TCR activation pathway is kept intact in μG, including at points both upstream and downstream of PKC.

#### 3.1.2. Ras/AP-1 Pathways

The Ras/AP-1 signaling cascade is another interesting pathway that may affect T cell activation and immune activity through the regulation of IL-2 and other activation-related cytokines. The AP-1 transcription factor is made up of c-jun and c-fos proteins complexed together and has similar functions to the NF-kB factors. AP-1 can be activated and translocated by the Ras/Raf/MEK/ERK signaling cascade, Ca^2+^ influx, and also by the signaling molecule PKC, discussed earlier. Specifically, the Ras and Ca^2+^ pathways lead to c-fos, and PKC leads to c-jun; together they form AP-1 [119]. These activation pathways may be gravisensitive, and it is important to understand them in order to properly define the dampening of T cell activation in μG.

In a study by Morrow, it was shown that nuclear levels of active AP-1 transcription factor were downregulated in 2-D clinorotation for human isolated T cells [120]. Specifically, it was mentioned that simulated μG may inhibit the binding of the transcription factors. In addition, the study supports the earlier conclusion that dysfunction occurs upstream of PKC, showing this time that AP-1 levels were diminished in μG when stimulated by anti-CD3/anti-CD28 but not when stimulated by PMA/ionomycin, which skips the early TCR proximal signaling molecules. Morrow further posits that the likely early signal dysfunction near the cell membrane has some relation to the changes in the cytoskeleton. It may be hypothesized that the interaction of cytoskeletal elements with TCR, PLC-γ, DAG, and other early activation molecules is disrupted due to μG -induced load changes.

As AP-1 is made from c-jun and c-fos, it is important to understand which of these, if not both, are responsible for the decreased AP-1. In the same study, Western Blot results showed that c-fos production was diminished in μG, showing that there is a gravisensitive step in either the Ras or Ca^2+^ pathways [120]. In a 72-h RWV experiment by Li et al., T cells stimulated by Con-A showed decreased levels of c-fos and NF-kB in the μG condition compared to experimental controls [90]. For c-jun, activation levels were unchanged in PMA-stimulated and anti CD3/CD28 T cells cultured in 2-D clinostat compared to controls [50]. To this end, it can be concluded that c-fos may be decreased in μG, and therefore may lead to diminished AP-1. However, c-jun may or may not be affected by μG. More experiments should be done to further elucidate the levels of activation in both of these proteins, especially c-jun.

Interestingly, an important regulator of c-jun activity is JNK, a MAPK enzyme that phosphorylates/activates c-jun [121]. In a 30-day spaceflight study, unstimulated mouse T cells were observed to have decreased levels of JNK in the real μG condition [85]. This might indicate that the levels of JNK and other regulatory immune molecules in an unstimulated in vivo environment are generally decreased, which hampers the output of the activation cascade once stimulated. Specifically, here, reduced levels of JNK before stimulation would in turn reduce the overall phosphorylation of c-jun, leading to impaired AP-1 activity. This hypothesis should be confirmed by studies aimed at directly measuring the levels of c-jun in μG. While the upstream pathway of c-jun has already been elucidated earlier in this paper (PKC), the pathways upstream of c-fos have yet to be investigated. One of those pathways involves the Ras/Raf/MEK/ERK cascade. In the same paper by Li et al., the level of phosphorylated (active) ERK1/2 in T cells stimulated by ConA in simulated μG demonstrated a significant decrease compared to the 1G control [90].

Continuing to investigate the activation of ERK, Paulsen et al. observed that ERK phosphorylation levels were increased in 5 min of RWV culture compared to 1G controls when stimulated by PMA or anti-CD3/anti-CD28 [50]. Another study by Simons et al. observed that ERK phosphorylation was unchanged between RWV and control when stimulated by anti-CD3/anti-CD28 [118]. Interestingly, Elk (another important activation transcription factor downstream of the ERK pathway) was also found to be unchanged in the stimulated RWV μG group compared to controls. These data are inconsistent with the data from Li et al. It is important to note the amount of time that the cultured cells were subjected to μG; though not well studied or controlled among different scientific papers, immune activity can show stark differences between short and long exposure to μG. It can be hypothesized that immune cells perceive acute μG differently from chronic exposure, and subsequently react in different manners. Therefore, the results about ERK may be taken by themselves, but cannot be compared to give an overall impression of the activity of ERK in μG.

Further upstream of ERK are the signaling proteins MEK and Raf. In the above study by Paulsen et al., it was observed that phosphorylation of MEK was enhanced 1.5-fold in PMA stimulated T cells under μG compared to 1G control. There was no change observed when groups were stimulated by anti-CD3/anti-CD28. In the same study, there was no observed change in the phosphorylation of Raf by either stimulation type in μG compared to control [50]. It can be concluded that there is still much uncertainty about the signaling molecules in this pathway and how they react to μG. Further research should aim to investigate the specifics of the Ras/AP-1 pathway in μG. The transcription factor CREB should also be mentioned, as it is an important factor in T cell activation [122]. CREB is a mediator of many of the activation pathways, and is observed to be phosphorylated when the T cell is activated. In a previously discussed article, it was found that the activation of CREB by phosphorylation was diminished in T cells in μG treated with Con-A and anti-CD28 compared to activated controls [67]. This indicates that CREB may be affected by a gravisensitive step, and should be researched further to fully understand its dysfunction in μG. The overall effect of microgravity on enzyme kinetics may also play a role in the dysregulation of these pathways, as they are predominantly regulated by kinase and phosphatase activity.

#### 3.1.3. Calcineurin/NFAT Pathways

Another important transcription factor involved in T cell activation is NFAT, usually activated through the Ca^2+^ influx/ calcineurin pathway. In the earlier study by Morrow, it was found that NFAT dephosphorylation (activation) remained intact in simulated μG. It was also found that NFAT binding to DNA was impaired, along with the binding of the other activation-related transcription factors [120]. The study postulates, based on the results showing active AP-1 being downregulated in μG, that NFAT activation remains intact but cannot bind to the DNA site because it is dependent on active AP-1 [123]. In the frame of decreased activation, this conclusion makes sense and is expected, but more experiments on NFAT are required to properly conclude about its specific activity in μG.

NFAT is the downstream target of the Ca^2+^ pathway (specifically calcineurin), and is therefore directly impacted by the influx of Ca^2+^ released from the endoplasmic reticulum. This release is normally triggered by IP3, which along with DAG is a product of the cleavage of PIP2 in the early TCR-proximal cascade [124]. In a study by Risso et al., T cells activated by anti-CD69 in μG provided by 2D clinostat showed increased levels of Ca^2+^ compared to the unstimulated group after 3 h of μG [88]. This is a positive indication of Ca^2+^ influx remaining intact in μG conditions. In a 72-h RWV study, T cells activated by PHA and/or ionomycin for 4 h showed increased levels of Ca^2+^ compared to unstimulated controls [66]. Similarly, another study concluded that the Ca^2+^ influx remains intact in simulated μG [65]. Taken together, there is evidence that the Ca^2+^ activation pathway from the point of Ca^2+^ influx may not be gravisensitive.

Through a thorough, stepwise analysis of the T cell activation signaling cascade, we can conclude that certain key gravisensitive steps significantly impair the outcome of the activation pathway. Working backwards from the main proinflammatory activation outputs and their transcription factors (such as NF-kB, AP-1, and NFAT), we can focus on the molecules of interest based on upstream and downstream activity. Many of the molecules going upstream of NF-kB transcription factors maintain their activity in μG up until PKC, shown by the studies using PMA to directly stimulate PKC. Similarly, c-jun (a part of AP-1) is also PKC moderated, though it may be that mediators such as JNK could be the gravisensitive culprit. AP-1’s other half, c-fos, is activated by the Ras/Raf/MEK/ERK pathway, but it is unclear how it is affected by μG. NFAT is different in that its activation alone does not seem to be affected by μG, and its upstream pathway from the Ca^2+^ influx remains largely intact. NFAT is dependent on AP-1, so the latter’s downregulation causes both to be unable to transcribe the activation-related genes needed.

The dysfunctional ends of these pathways largely point to the cascade upstream of PKC, after TCR itself. One of these molecules is PIP2 which is hydrolyzed to produce IP3 and DAG, which in turn signal the Ca^2+^ and PKC pathways, respectively. Having evidence that Ca^2+^ influx and its pathway remain intact in μG, it likely indicates that the hydrolysis of PIP2 into IP3 and DAG also may remain intact. Several other molecules that make up a cascade network proximal to the TCR include ZAP-70, PLCy1, SLP-76, and others. These membrane-proximal molecules may easily be influenced by the documented cytoskeletal dysfunction in μG. Therefore, we propose that the key gravisensitive step may occur in the DAG-mediated activation of PKC or the early-cascade TCR-proximal molecules. Furthermore, the production of IP3, DAG, PIP2, PIP3, and other inositol molecules may be an important consideration for future studies given their proximity to the cell surface. A special effort needs to be made to uncover the exact mechanistic dysfunction that occurs in these steps.

Another well-studied signaling molecule is IFN-γ, an important mediator of the human immunological response. IFN-γ is the primary in vivo activating molecule for macrophages, and is usually produced by T cells and NK cells upon their activation. The IFN-γ signal proceeds along the JAK-STAT pathway, inducing gene expression relating to NO production, MHC class II production, and more [125]. It is an important output molecule for T cells that helps bind the adaptive and innate immune responses into a complete and unified defense. Therefore, keeping its importance as a T cell activation product and immune regulator in mind, it should be studied as a molecule of interest when investigating immune dysfunction in μG.

In a 2015 study by Crucian et al., blood samples were collected from 23 astronauts on a six month ISS mission in order to study the impairment of adaptive immunity in long-duration spaceflight. The in-flight samples displayed significantly decreased levels of IFN-γ upon T cell stimulation, among other cytokines as well [84]. This is an indicator that downstream immune function is significantly impaired in real μG situations. In a 45-day human subject HDBR study by Xu et al., it was observed that plasma IFN-γ levels (measured by cytometric bead array analysis) gradually decreased over time in the simulated μG condition, with the lowest levels on the final day [45]. In a rotary bioreactor (RWV) experiment, mouse splenocyte T cells that were stimulated with Con-A or PMA/ionomycin after pre-exposure to simulated μG showed decreased levels of IFN-γ expression [64]. Similarly, in an experiment on Con-A/anti-CD28 activated human T cells, IFN-γ gene induction was suppressed in simulated μG via RPM [67]. In a recent study by Spatz et al., T cells cultured in an RWV and stimulated by Con-A/anti-CD28 for 1.5 h exhibited markedly lower expression of IFN-γ in μG compared to 1G control, further establishing the downregulation of the pathway outcome [58]. Interestingly, at 4 h of stimulation, the IFN-γ expression was not significantly altered, but decreased overall compared to the 1.5-h control; this might indicate a desensitization or saturation of the cells to the stimulant, leading to insignificant IFN-γ findings. Several other studies show similar results in μG IFN-γ assays [15,46,67,69,82,85,94,102,104,126].

It should be noted that there are also several studies that contradict this finding, instead observing that T cell-produced IFN-γ is increased or unchanged in spaceflight or simulated μG conditions [57,75,85,90,96,98,100,105]. This finding is unusual, as it goes against the general observation of impaired pro-inflammatory response in μG. Many of these studies concluded that IFN-γ production can be influenced by stimulation type, stimulation time, cell line, post-flight vs. in-flight collection, and other factors that might explain the contradictory finding. Overall, IFN-γ plays an important part in the downstream T cell activation immune response and is generally considered to be significantly affected by μG, which in turn would affect adaptive and innate human immunity as a whole.

### 3.2. Signaling Pathways Responsible for T Cell Apoptosis under Microgravity

Apoptosis has many homeostatic purposes in cells of the human body [26,127]. In the immune system, programmed cell death is important for immune response, tumor suppression, and cytotoxic killing [128]. It has been observed that apoptosis is significantly increased in T cells subjected to μG [78,85,89,92,101,129,130,131,132,133]. Fas/APO-1 (also known as FasR or CD95) is a cell surface receptor and is an important regulator of apoptosis in T cells, and is studied because it is the best-defined factor of apoptosis signaling [129]. The interaction of Fas and Fas ligand (Fas-L) constitutes the beginning of the apoptotic pathway. In a study of OVA-stimulated mouse splenocyte T cells subjected to real μG during spaceflight, there was a larger number of cells in the ground control versus spaceflight [101]. To investigate the presence of apoptotic upregulation, Fas-L relative expression was measured and observed to be increased in the μG treated group. This suggests that an increase of Fas-L correlates to more apoptosis in-flight versus 1G ground when unstimulated (though stimulated cells did not have a significant enough change).

In a similar experiment that utilized a space shuttle to provide real μG to Jurkat T cells, it was observed that apoptosis was markedly increased compared to 1G controls [129]. By utilizing DNA condensation staining, it was found that there was 28% apoptosis in μG and only 12% apoptosis in ground control. This suggests that apoptosis is significantly upregulated (over two times as much) because of μG. To verify the involvement of Fas/APO-1, an ELISA kit was used to measure expression levels at different time increments. It was observed that Fas expression was increased by 15× between 4 and 24 h, and 65× at 48 h. This proves that Fas is significantly increased in a time-dependent manner in μG. Putting the results of this study together, we can conclude that the increase in Fas likely correlates with the upregulated apoptosis when subjected to μG.

Increased levels of Fas/CD95 were further exhibited as a result of modeled μG in CD8+ T cells, stimulated by Con-A in a RWV experiment [64]. Also, late apoptotic CD4+ and CD8+ T cells were observed to increase in μG compared to controls. Although the significance of CD4+ T cells was not established for the Fas expression assay, the overall data corroborates the proposition that the Fas signaling step of apoptosis is directly affected by μG. In another study, T cells subjected to μG via spaceflight and activated by increasing FBS concentration and temperature showed an increase in Fas levels and subsequent apoptosis [130]. Specifically, the number of Fas positive cells was increased in μG at 48 h compared to ground samples, tested by immunofluorescence microscopy. Also, Fas levels were expectedly time-dependent and increasing in μG. The percent of apoptotic cells also increased significantly in μG as compared with ground controls, correlating with Fas expression levels. Interestingly, this study also showed that cell density was not a factor in determining cell viability/apoptosis in space-flown cell cultures, strengthening the argument that μG impairs cell viability by inducing apoptosis. Based on these studies, it can be reasonably said that Fas/APO-1 receptor (CD95) is upregulated in T cells under real and simulated μG and leads to an increase in apoptosis [129,130,131]. We should thus shift the investigation to what causes the increased levels of these (and other) apoptotic molecules.

**Figure 3:**
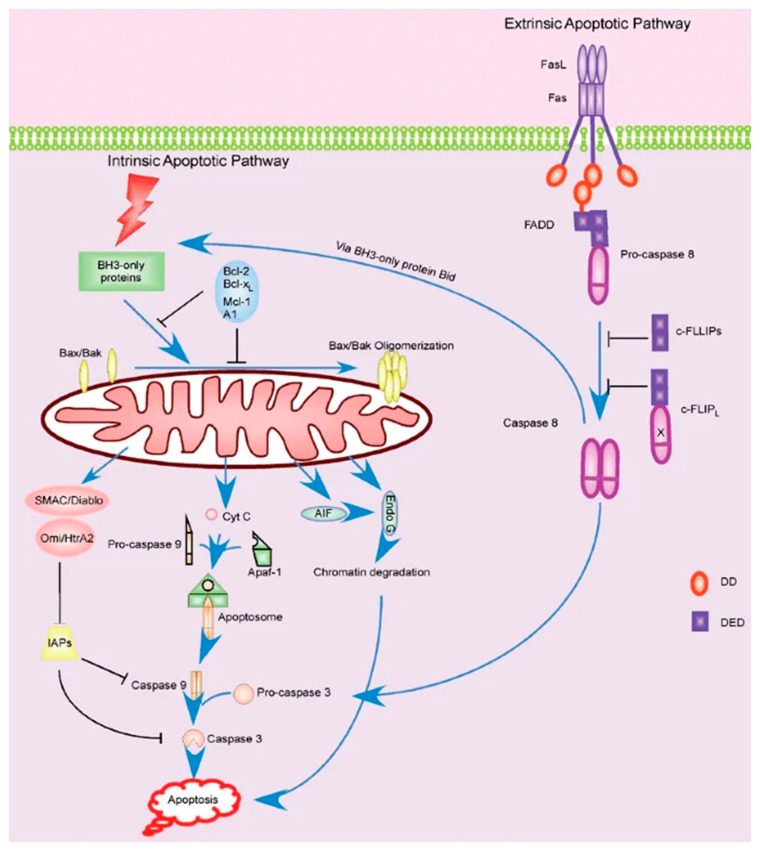
Extrinsic and intrinsic apoptotic pathways within the T cell [134].

Apoptotic signaling is broken into a few notable pathways, as shown in Figure 3. Firstly, the Fas-L mediated pathway (known as the extrinsic pathway) is immediately responsible for inducing apoptosis by signaling other cells and self-signaling, as noted earlier [134]. However, the signaling cascade that leads to the expression of Fas in the first place should be our primary interest. Fas-L is thought to be produced as a result of over-activation as a means of shutting down hyperactive cells, though Fas/APO-1 is constitutively expressed in activated T cells [129]. Alternatively, the apoptotic cascade can be initiated by the intracellular (intrinsic) pathway. This pathway is sparked by the cell’s perception of stress, be it by physical damage, radiation, heat, or other processes. This pathway is normally inhibited by Bcl-2, and suppression of this protein can lead to uncontrolled or increased apoptosis [135].

Bcl-2 is one of the most important modulators of the apoptotic pathway. It is normally present in high levels in actively working cells. Apoptosis can be induced if Bcl-2 is suppressed in some manner. Sokolovskaya et al. investigated the behavior of Bcl-2 in T cells under μG via RPM. They observed that Bcl-2 remained unchanged in the μG group of non-activated Jurkat T cells compared to the 1G controls [131]. In a similar study, anti-CD3 activated T cells in a RWV also showed that Bcl-2 was unchanged in μG compared to 1G [136]. The study also measured the levels of BAX, which along with BAK, is an important pro-apoptotic molecule along the intrinsic pathway. It was observed that these levels did not change in the μG condition. It is important to note that Fas and Fas-L were both also unchanged in this same study, which contradicts many of the previous studies and may invalidate part of the results. In another RWV study, it was shown that both Bcl-2 and BAX were downregulated in T cells under μG conditions [137]. Taken as a whole, it may be possible that the intrinsic pathway is not significantly affected in any meaningful way by μG.

Apoptosis can be an outcome of cell stress if the perceived damage is too great, but cells have mechanisms in place to survive through some stressors. One of the most important of these mechanisms is Heat Shock Protein (HSP). HSPs are a family of proteins that can activate signaling cascades and cell functions when they sense the cell is under stress, including helping in protein folding, chaperoning, and cell proliferation [138]. HSPs also inhibit bid and cytochrome c, which are both pro-apoptotic factors [26]. In a study by Novoselova et al., it was observed that HSP-72 and HSP-90 were both increased in non-activated mouse T cells after 12 h and 7 days in spaceflight, respectively, compared to ground controls [85]. However, it should be noted that only postflight samples were taken, which does not control for take-off and landing stress. In another HSP study, it was found that HSP-70 was significantly increased, but HSP-90 was significantly decreased [27]. It is expected that all HSPs should increase due to stress; however, the details of microgravity-induced cellular stress are relatively unknown. The study suggests that μG may have a very specific effect on HSPs individually. Additionally, it was observed that HSP-70 remained unchanged in the previously discussed study by Sokolovskaya et al. [131]. Taken together, there is conflicting evidence about the effect of μG and how it influences the levels of HSPs. More research should be done to further elucidate the behavior of HSP regulation in μG.

Although apoptosis is an important method of immune system (specifically T cell) dysfunction in μG, it has not been as well studied as its importance may suggest. There has yet to be conclusive evidence exposing the mechanisms behind increased apoptosis, other than the increase in Fas/APO-1 expression. However, the observation that apoptosis in T cells is increased in μG is well established in itself. We posit that due to this increase in cell death and subsequent decrease in viable cells in μG conditions, testing for activation of T cells may be easily skewed. Many activation-related studies do not control for an increase in apoptosis, thereby potentially decreasing the number of activation products and implying that the activation suppression is worse than if it were just due to cell signaling disruption. To this end, it is crucial to control for both apoptosis and activation in experiments in order to allow for a greater understanding of overall T cell behavior in μG.

## 4. Future Directions

The future of space aviation and exploration hinges heavily on the biomedical research conducted prior to long-duration missions. Maintaining astronaut health starts with rigorous and organized scientific experimentation. When conducted properly, the research surrounding immune dysregulation in μG may uncover the mechanisms needed to develop countermeasures and treatments that support astronaut well-being and contribute to space exploration. Given that adaptive immunity varies widely from person to person, it is important to develop personalized countermeasures to target and combat T cell dysfunction. In order to conclude and draw from this research, we propose organized and diligent experimental standards for conducting μG research in relation to immune function.

Regarding methods of exposing biological samples to μG, it is important that researchers take into account the benefits and drawbacks of each method with regard to the specific experiment, both real and simulated. It is important to establish a reason to use that method, and then choose parameters that align with the aim of the study. Each type of μG exposure may yield slightly different results, hence the importance of controlling for many factors and of comparing methods with similar experimental parameters. Of the studies reviewed here, the experimental design and parameters were often the most inconsistent and different among similar studies.

It is important to note the time that cells are exposed to μG, the time of activation, activation type, the order of methods, and other factors. T cells and other immune cells may act differently under acute and chronic μG exposure; the focus of the study should determine the timings. To be clinically impactful, studies should aim to be longitudinal, taking measurements at both short and long time period points in order to accurately convey data that shows how expression changes throughout μG exposure. Activation time may depend on the stimulation method but should generally be controlled and cross-referenced with activation marker expression in control groups. Similarly, many studies were inconsistent in whether activation was introduced before, during, or after μG exposure. A focused study should give justification as to why this order was chosen, and why that data is significant for clinical purposes.

One of the most important control factors that many real μG studies did not incorporate is a 1G control group on-board the spacecraft. Takeoff and landing account for significant homeostatic and cellular stress, subjecting the human body to large G forces. To control for these factors, simulated 1G gravity provided by centrifuge or other means should be used on samples on board the spacecraft, and compared to both the experimental groups and the ground control group. This way, data is much more significant and large sources of error may be avoided.

Regarding specific T cell activation, further research needs to be done to fully elucidate the mechanism behind its dysfunction. Using stepwise analysis of the current literature, we observed that many of the downstream signaling functions affected by μG seem to remain intact when stimulated directly. These observations lead us to believe there may be a gravisensitive step involving a TCR/membrane-proximal signaling molecule. We posit that future research should specifically target these early cascade molecules and empirically determine any significant changes due to μG exposure. Furthermore, knowing that these molecules may integrate or interact with the cell cytoskeleton, it would be wise to pair these findings with those of cytoskeletal studies investigating mechanistic changes due to μG. Recently, a special mechanosensitive family of receptors, known as Piezo receptors, have been a topic of interest regarding T cell activation [139]. As they are direct mechanoreceptors, it may be worthwhile to investigate how these receptors react to a microgravity environment and their downstream effects. It is possible that any interconnected findings could explain active T cell suppression in μG.

When conducting experiments or analyzing data, it is important to note that changes in expression of proteins must always be normalized using the sample population. This prevents the decreased proliferation and number of cells in microgravity from skewing the data. Not all studies report appropriate normalization to show alterations in protein levels with respect to cell population and viability. Similarly, studies focusing on apoptotic changes in T cells must have their own proper controls. While the method of apoptosis upregulation in μG is still unknown, research efforts should be directed toward finding which pathways are gravisensitive. As stated earlier, it is important to control for both apoptosis and activation during any study, as either may affect the other. Increased cell death may skew activation and proliferation data if not controlled and accounted for, and is therefore important when consider in any future immune cell μG experiments. To understand the interaction of the immune response with the stress of microgravity, future experiments must focus on specific signaling cascades, and systematically investigate each step to determine the gravisensitive structures and proteins that give way to cellular impairment. Overall, the future of this field depends on collaborative and strategic efforts to control studies, parameters, and experimental design so as to properly uncover the effect of μG on immune cell function.

## 5. Conclusions

Identification of the mechanisms behind immune dysfunction in μG is extremely relevant to space exploration. Such mechanisms can lead to specific countermeasures and treatments aimed at preventing adverse physiological responses in astronauts. Preserving the health of astronauts is a necessity if humans are to embark on a long-duration space mission. Alongside the immune system, understanding how the human body reacts in space overall may directly affect the future of space exploration. With regard to immunity, this review has explored in-depth the effect of μG on adaptive immunity, with specific attention to T cells. It is established that T cells show both diminished activation via decreased pro-inflammatory cytokines such as IL-2, and increased cell death observed by cell viability assays and increases in Fas/Fas-L. A systematic review of the signaling pathways involved in the processes yields much information but stops short of identifying exact gravisensitive steps using current literature. In activation pathways, outputs, and downstream molecules are potentially impacted by early signal, membrane-proximal molecules. These likely include molecules upstream of PKC and downstream of the TCR. There is a plethora of molecules in the complex interconnected system of activation signaling and some steps within may show evidence of alteration in μG, including the Ras/AP-1 and Calcineurin/NFAT pathways. Cell death due to apoptosis may be affected by a multitude of pathways that sense gravity alteration as an extracellular stressor. Taken as a whole, specific, goal-focused, collaborative, and controlled research needs to be implemented in the future to fully understand the complex inner workings of immune gravisensitive dysregulation in space. The development of preventative treatments that target T cell signaling can help maintain astronaut health in space and sustain the goals of human space exploration.

## Figures and Tables

**Table 1:** A compilation of T cell related pro-inflammatory and anti-inflammatory cytokines in μG experiments organized by the least to the most advanced method of μG exposure and duration of the experiment. T cells were taken from human blood, peripheral blood mononuclear cells (PBMC), mice, rats, Jurkat T cells, and OTII-TCH cells. Single-headed arrows (↓↑) indicate an increase or decrease, double-headed arrows (↟↡) indicate a significant increase or decrease, and slashes (/) indicate no change.

			Pro-Inflammatory									Anti-Inflammatory					
Microgravity Exposure	Time	T Cell Source	IL-1B	IL-1	IL-2	IL-5	IL-12	IL-17/A	IL-22	IFN-y	TNF-a	IL-4	IL-6	IL-8	IL-10	TGF-B1	Study
Headdown Bedrest	21 days	Human blood			↡					↡	↡				↡		[46]
	45 days	Human blood			/			↡	/	↡	/	/	/		↑	↑	[45]
	120 days	Human blood											↟				[44]
2D Clinostat	12 h	PBMC		↑	↓												[88]
	48 h	PBMC			↡												[68]
	48 h	PBMC			↡												[68]
Rotary Wall Vessel	3.5 h	Mice			↡					↡							[69]
	18 h	PBMC								/	↡						[58]
	24 h	Rats			↡					↡							[64]
	24 h	Rats			↡					↡							[64]
	48 h	PBMC					↟										[62]
	48 h	Jurkat T Cells			↡					↟		↡	/		/		[89]
	3 days	PBMC	↑		↓					↓			↑				[66]
	72 h	Rats			↡												[90]
	72 h	PBMC			↓					/							[57]
	72 h	PBMC										↡					[27]
	72 h	Mice			↡												[61]
	120 h	OTII-TCH cells			↓												[91]
3D Clinostat	4 h	PBMC			↡					↡	↡						[67]
	12 h	PBMC			↡												[92]
	12 h	PBMC		↑	↓												[68]
	24 h	PBMC			/					↡	↟				/		[71]
	26.5 h	Mice			↡					↡							[69]
Parabolic Flight	48 h	PBMC			/						/		/		/		[76]
Spaceflight	6 h	PBMC			/					↑							[75]
	14 h	Jurkat T Cells		↓	↓												[93]
	72 h	PBMC		↟	↡					↡	/						[94]
	4 days	Rats			/												[95]
	9 days	Mice								↟							[96]
	10 days	Human blood									↡						[80]
	10 days	Mice			↓												[97]
	13 days	Mice			/					/	↟		/				[98]
	13 days 19 h	Mice			/												[99]
	14 days	Human blood			↟	↡		/		↡	↡	↡	↡		↡		[100]
	14 days	Human blood			/	↡		/		↡	↡	↡	↡		↡		[100]
	14 days	Human blood	/				/				/		/	↟	/		[100]
	15 days	Mice	↟		↡			↟		↑	↑	/	↑		↑		[101]
	15 days	Mice			↡					↡							[69]
	18 days	Human blood			↡					↡							[102]
	30 days	Mice								↡	/		↡				[85]
	175 days	Human blood			/					/							[103]
	180 days	Human blood								/	/		↟				[104]
	6 months	Human blood			/	↡		↡		/	/	↡	/		↡		[84]
	6 months	Human blood			↓	↡		↡		↡	↡	↡	↡		↡		[84]
	6 months	Human blood	↓								/		/	↑	↓		[84]
	11 months	Human blood			/					/							[105]

## Data Availability

The data presented in this study are available within this article.
